# Porcine adipose-derived stem cells from buccal fat pad and subcutaneous adipose tissue for future preclinical studies in oral surgery

**DOI:** 10.1186/scrt359

**Published:** 2013-12-11

**Authors:** Stefania Niada, Lorena Maria Ferreira, Elena Arrigoni, Alessandro Addis, Marino Campagnol, Eugenio Broccaioli, Anna Teresa Brini

**Affiliations:** 1 Dipartimento di Scienze Biomediche, Chirurgiche e Odontoiatriche, Università degli Studi di Milano, Via Vanvitelli, 32, 20129 Milan, Italy; 2 IRCCS Istituto Ortopedico Galeazzi, Milan, Italy; 3 CRABCC srl, Rivolta d’Adda, Cremona, Italy

## Abstract

**Introduction:**

Adipose-derived stem cells (ASCs) are progenitor cells used in bone tissue engineering and regenerative medicine. Despite subcutaneous adipose tissue being more abundant, the buccal fat pad (BFP) is easily accessible for dentists and maxillofacial surgeons. For this reason, considering the need for preclinical study and the swine as an optimal animal model in tissue engineering applications, we compared the features of porcine ASCs (pASCs) from both tissue-harvesting sites.

**Methods:**

ASCs were isolated from interscapular subcutaneous adipose tissue (ScI) and buccal fat pads of six swine. Cells were characterized for their stemness and multipotent features. Moreover, their osteogenic ability when cultured on titanium disks and silicon carbide-plasma-enhanced chemical vapor-deposition fragments, and their growth in the presence of autologous and heterologous serum were also assessed.

**Results:**

Independent of the harvesting site, no differences in proliferation, viability, and clonogenicity were observed among all the pASC populations. Furthermore, when induced toward osteogenic differentiation, both ScI- and BFP-pASCs showed an increase of collagen and calcified extracellular matrix (ECM) production, alkaline phosphatase activity, and osteonectin expression, indicating their ability to differentiate toward osteoblast-like cells. In addition, they differentiated toward adipocyte-like cells, and chondrogenic induced pASCs were able to increase glycosaminoglycans (GAGs) production over time. When cells were osteoinduced on synthetic biomaterials, they significantly increased the amount of calcified ECM compared with control cells; moreover, titanium showed the osteoinductive effect on pASCs, also without chemical stimuli. Finally, these cells grew nicely in 10% FBS, and no benefits were produced by substitution with swine serum.

**Conclusions:**

Swine buccal fat pad contains progenitor cells with mesenchymal features, and they also osteo-differentiate nicely in association with synthetic supports. We suggest that porcine BFP-ASCs may be applied in preclinical studies of periodontal and bone-defect regeneration.

## Introduction

Dental tissue engineering may now represent an innovative approach to replacing bone and periodontal ligament lost, through the delivery of bioactive molecules and the use of suitable scaffolds and cells. Advanced research in this field leads to rapid progress in tissue repair and regeneration of oral tissues. Mesenchymal stem cells (MSCs), because of their ability to self-renew, their multidifferentiative potential toward mesodermal cells [1-3], and their plasticity toward cells of ectodermal [4] and endodermal [5,6] origin, are considered proper candidates for these applications. Bone marrow is still the elected source for MSCs [7], although adipose tissue, in the last decade, gained recognition, because adipose-derived stem cells (ASCs) can be easily extracted with mild donor-site morbidity or patient discomfort [8]. The first ASCs were isolated from subcutaneous adipose tissue, which is usually discarded after aesthetic surgical procedures. Several studies have also described the presence of ASCs in visceral adipose tissue [9], human orbital fat tissue [10,11], and from special fat pads such as the Hoffa pad [12].

Here, we propose the buccal fat pad (BFP) as a new source for ASCs, which could be of great interest for odontoiatric and maxillofacial surgeons who consider the tissue-engineering approach to be a possible future goal.

The BFP is located between the masseter and buccinators muscles and the ascending mandibular ramus and zygomatic arch [13], and it is easily accessible with a simple surgical procedure under local anesthesia [14]. Since 1977, the BFP has been used in surgery for the treatment of congenital oroantral and/or oronasal diseases [15], congenital cleft palate repair [16], oral submucous fibrosis [17,18], intraoral malignant defects [19], and cheek mucosa defects [15,20]. In addition, BFP is a discarded tissue of plastic surgery for cheek reduction. Recent studies showed that human ASCs isolated from the BFP possess all the suitable characteristics for bone tissue engineering, both *in vitro*[21] and *in vivo*[22]. Despite the known low immunogenicity of human ASCs, which suggests theoretically their use in preclinical models, we are required to test their safety when implanted in a homologous setting. Considering the potential ability of ASCs in bone regeneration, we have chosen swine as a preclinical model because their bone shares several features with the human bone, such as rate of healing, morphology, anatomy [23], mineral density, and composition [24]. Furthermore, the oral maxillofacial region of these animals is similar in anatomy, development, physiology, pathophysiology, and disease occurrence to the human one [25]. Therefore these animals might be considered appropriate for oral disease models and in orofacial research; they were recently used in preclinical models of dental implants [26-28] and maxillofacial surgery [29-31].

Despite the great variety of supports used in tissue engineering, titanium is widely used in dental surgery, due to its high mechanical and corrosion resistance, as well as its biocompatibility. Interestingly, silicon carbide (SIC), with its hardness and wear-resistance, may be an innovative material suitable to coat metallic implants, giving adequate protection to the material and decreasing the wear rate of the inserted devices. Moreover, SIC obtained by the plasma-enhanced chemical vapor-deposition technique (SIC-PECVD) does not negatively influence any biologic features of human ASCs, *in vitro*[32], and SIC particles do not give rise to any relevant inflammatory response and do not negatively affect bone growth *in vivo*[33].

In this study, we described some of the features of ASCs isolated from swine BFP and interscapular subcutaneous adipose tissue from the same animal, and their osteodifferentiation ability *in vitro,* either in the absence or in the presence of titanium and SIC supports. Finally, pASCs cultured in the presence of autologous and heterologous serum have been also investigated.

## Materials and methods

### Isolation of porcine adipose-derived stem cells (pASCs)

Fat tissues and blood samples were collected at the end of preclinical studies approved by the Italian Ministry of Health and were performed at the CRABBC (Biotech Research Centre for Cardiothoracic Applications) (Rivolta d’Adda, CR). All the procedures were carried out in conformity with institutional guidelines in compliance with national (Law 116/92, Authorization n.169/94-A issued December 19, 1994, by the Italian Ministry of Health) and international laws and policies (EEC Council Directive 86/609, OJ L 358. 1, December 12, 1987).

**Figure 1:**
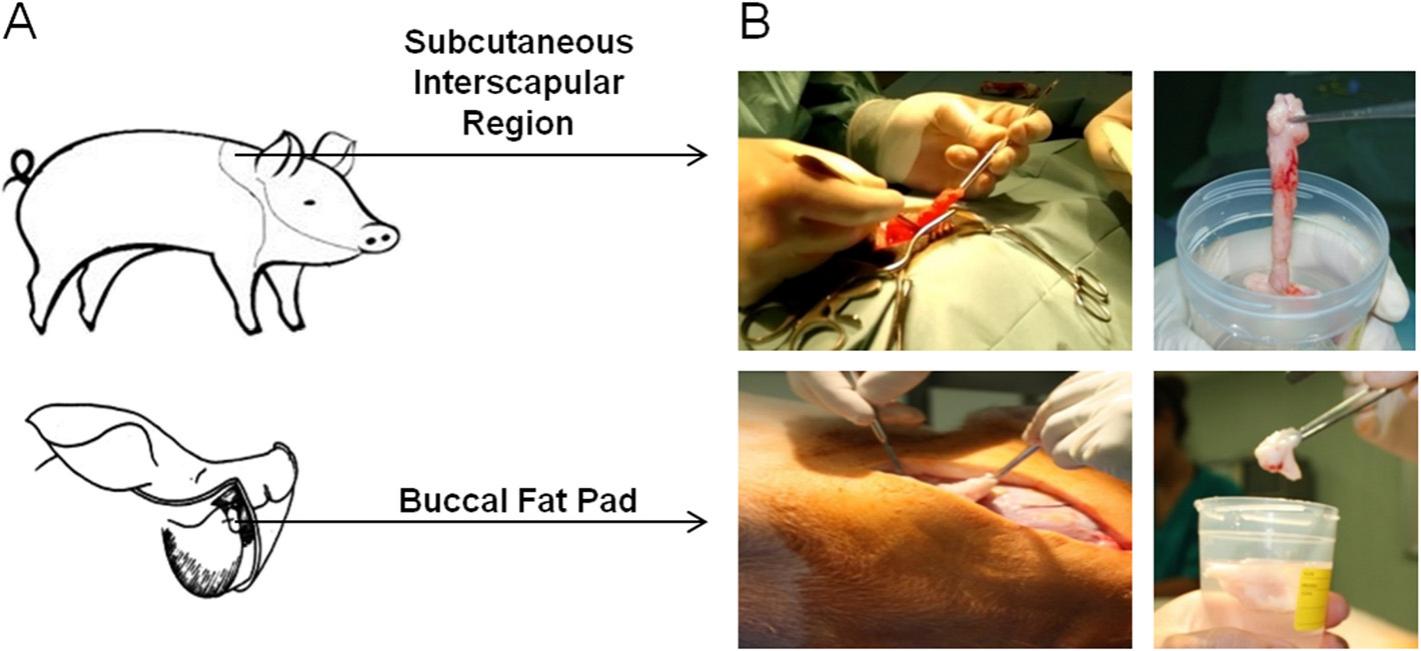
**Localization of subcutaneous interscapular and buccal fat pad tissue withdrawal.** Anatomic regions of subcutaneous interscapular adipose tissue and buccal fat pad **(A)**. Surgical procedure for tissue collection **(B)**.

Adipose tissues were collected from subcutaneous interscapular sites (ScIs) and buccal fat pads (BFPs) (Figure 1A, B) from six swine. Porcine adipose-derived stem cells (pASCs) were isolated as previously described. In brief, tissues were enzymatically digested with 0.1% type I collagenase (225 U/mg; Worthington, Lakewood, NJ, USA) at 37°C for 60 minutes. The stromal vascular fraction (SVF) was centrifuged, filtered, and 10^5^ cells/cm^2^ were plated in DMEM (Sigma-Aldrich, Milan, Italy) supplemented with 10% FBS, 50 U/ml penicillin, 50 μg/ml streptomycin, and 2 m*M* L-glutamine (Sigma-Aldrich) (control medium, CTRL). Cells were maintained at 37°C in a humidified atmosphere with 5% CO_2_. When cells reached 70% to 80% confluence, they were detached with 0.5% trypsin/0.2% EDTA (Sigma-Aldrich) and plated at a density of 5 × 10^3^ cells/cm^2^.

### Proliferation

About 5 × 10^3^ cells/cm^2^ were maintained in culture for three passages, and regularly detached and counted. Proliferation rate was expressed as doubling time (DT), calculated as follows: ln(N/N_0_)/ln_2_, in which N represents the number of counted cells, and N_0_ represents the number of seeded ones.

### MTT cell-viability assay

To test the viability of cells, 1.5 × 10^4^ pASCs/cm^2^ were plated in 96-well plates, and monitored at days 1, 3, and 7. Then 100 μl of MTT (3-[4,5 dimethylthiazol-2-yl]-2,5-diphenyltetrazolium bromide; Sigma-Aldrich) (final concentration, 0.5 mg/ml in DMEM) was added, and cells were maintained for 4 additional hours at 37°C. Formazan precipitates were solubilized by 100% DMSO (dimethylsulfoxide; Sigma-Aldrich), and absorbance was read at 570 nm in a Wallac Victor II plate reader (Perkin Elmer Western Europe, Monza, Italy) [34].

### Fibroblast-colony-forming unit assay (CFU-F)

pASCs were plated in DMEM supplemented with 20% FBS, 50 U/ml penicillin, 50 μg/ml streptomycin, and 2 m*M* L-glutamine, in six-well plates by serial dilution starting from 1,000 cells/well. After 6 days, the medium was replaced, and, at day 10, cells were washed, fixed in 100% methanol, and stained with 0.5% crystal violet (Fluka, Buchs, Switzerland). The frequency of the CFU-F was established by counting individual colonies (of at least 25 cells) compared with the number of seeded cells.

### Flow-cytometry analysis

The pASCs (3 × 10^5^) in 100 μl of PBS with 1% FBS and 0.1% NaN_3_ were incubated for 30 minutes on ice with monoclonal antibodies raised against CD14, CD45, CD73, CD90, CD105, and CD271 (Ancell, Bayport, MN, USA). In particular, CD73, CD90, and CD105 were chosen according to the minimal criteria for defining mesenchymal stem cells [35,36]. Specific binding was revealed by either streptavidin-PE– or fluorescein isothiocyanate–conjugated sheep anti-mouse antibody. Samples were acquired by MACSQuant Analyzer (Miltenyi Biotec, Italy), and data were analyzed by using MACSQuantify Software (Miltenyi Biotec).

### Osteogenic differentiation

Cells were maintained in either control or osteogenic medium (OSTEO, DMEM, 10% FBS, 10 n*M* dexamethasone, 10 m*M* glycerol-2-phosphate, 150 μ*M* L-ascorbic acid-2-phosphate, 10 n*M* cholecalciferol; Sigma-Aldrich) in 24-well plates at the density of 2 × 10^4^, 1 × 10^4^, and 5 × 10^3^ pASCs/well for 7, 14, and 21 days, respectively.

After 7 and 14 days, to determine collagen production, cells were stained with 0.1% (wt/vol) Sirius Red F3BA in saturated picric acid (Sigma-Aldrich) for 1 hour at room temperature, and then the samples were extracted with 0.1 *M* NaOH for 5 minutes [37]. Absorbance was read at 550 nm, as previously. Standard curve of known concentration of calf-skin type I collagen (Sigma-Aldrich) was used to determine the concentration of secreted collagen.

Extracellular matrix (ECM) calcification, at 14 and 21 days, was determined on fixed ASCs stained by 40 m*M* Alizarin Red-S (AR-S, pH 4.1; Fluka). Mineral deposition was quantified by incubating the stained sample with 10% wt/vol cetylpyridinium chloride (CPC; Sigma-Aldrich) in 0.1 *M* phosphate buffer (pH 7.0) for 15 minutes to extract AR-S. Absorbance was read at 550 nm with a Wallac Victor II plate reader [38]. To evaluate alkaline phosphatase (ALP) enzymatic activity, both undifferentiated and differentiated ASCs were lysed in 50 μl of 0.1% Triton X-100 and incubated at 37°C with 10 m*M p*-nitrophenylphosphate dissolved in 100 m*M* diethanolamine and 0.5 m*M* MgCl_2_, pH 10.5. Samples were read at 405 nm, and ALP activity was standardized with respect to the sample protein concentration determined by BCA Protein Assay (Pierce Biotechnology, Rockford, IL, USA).

Osteonectin (ON) expression was also analyzed with Western blot: both undifferentiated and osteo-differentiated cells were lysed in 50 m*M* Tris pH 8, 150 m*M* NaCl, 1% Nonidet P40, 0.1% sodium dodecylsulfate (SDS), supplemented with protease inhibitor cocktail. Then 20 μg of protein extracts was resolved by 12.5% SDS-polyacrylamide gel (Bio-Rad Laboratories), electrotransferred onto HybondTM-ECLTM extra nitrocellulose membrane (GE Healthcare), and probed with either mouse anti-ON (1:100 dilution; Santa Cruz Biotechnology), and mouse anti-β-actin (1:5,000 dilution, Sigma-Aldrich). Specific proteins were revealed by horseradish peroxidase (HRP)-conjugated secondary antibodies (GE Healthcare) and the ECL Western Blotting Analysis System Kit (GE Healthcare), according to the manufacturer’s protocol.

### Adipogenic differentiation

Porcine ASCs were induced to differentiate toward the adipogenic lineage, as previously described [21]. In brief, 1.5 × 10^4^ pASCs/cm^2^ were plated and cultured in control medium supplemented with 1 μ*M* dexamethasone, 10 μg/ml insulin, 500 μ*M* 3-isobutyl-1-methyl-xanthine, and 200 μ*M* indomethacin (Sigma-Aldrich). At 14 days later, cells were fixed in 10% neutral buffered formalin for 1 hour and stained with fresh Oil Red O solution (20 mg/ml (wt/vol) Oil Red O in 60% isopropanol) for 15 minutes. Lipid vacuoles were quantified by extraction with 200 μl of 100% isopropanol for 10 minutes and reading the absorbance of 50 μl at 490 nm with the Wallac Victor II plate reader.

### Chondrogenic differentiation

Then 5 × 10^5^ pASCs were cultured in micromasses in chondrogenic medium (DMEM supplemented with 1% FBS, 100 n*M* dexamethasone, 110 mg/L sodium pyruvate, 150 μ*M* L-ascorbic acid-2-phosphate, 1× insulin-transferrin selenium (ITS) and 10 ng/ml TGF-β1) for 21 days. Glycosaminoglycans (GAGs) production was assessed with dimethylmethylene blue (DMMB) assay, as previously described [39,40]. In brief, micromasses were digested at 56°C overnight by 100 μl of 50 μg/ml proteinase K in 100 m*M* K_2_HPO_4_ (pH 8.0). After 10 minutes at 90°C to inactivate the enzyme, the samples were spun at 14,000 *g* for 10 minutes, and each supernatant was collected for GAGs and DNA quantification. The samples were then incubated at room temperature in 40 m*M* glycine/NaCl (pH 3) with 16 mg/ml DMMB, and the absorbance was read at 500 nm with the Wallac Victor II plate reader. The amount of GAGs was determined with respect to known concentrations of chondroitin sulfate (Sigma-Aldrich) and normalized on total DNA content determined as described later. Then 0.2 μg/ml Hoechst 33258 was added to the samples for 1 minute at room temperature, fluorescence was measured (excitation at 340 to 370 nm; emission, 440 to 460 nm), and DNA concentration for each sample determined with respect to the standard curve of salmon sperm DNA.

### ASC culture and osteogenic differentiation on biomaterials

Both ScI- and BFP-pASCs were seeded at 5 × 10^3^/cm^2^ on titanium disk (kindly provided by Permedica S.p.A., Merate, Italy) and silicon carbide–plasma-enhanced chemical-vapor deposition (SIC) fragments (kindly provided by CETEV, Centro Tecnologico del Vuoto, Carsoli, AQ, Italy) either in CTRL or OSTEO medium. To determine cells adhering to the biomaterials, both undifferentiated and differentiated pASCs for 21 days, were lysed in 0.1% Triton X-100, and protein concentration was determined by BCA Protein Assay, as described earlier. Meanwhile, in adjacent wells, calcified ECM deposition was determined, and compared with the one produced by plastic-adherent (PA) cells.

### Porcine serum collection

Then 10 ml of blood from each animal was allowed to clot for 30 to 45 minutes at 37°C and then transferred at 4°C for 30 minutes. After centrifugation (1,000 *g* for 10 minutes), sera were collected under sterile conditions [41] and maintained at -20°C until their use.

### Statistical analysis

Data are expressed as mean ± SEM. Statistical analyses were performed by using Student *t* test. Differences were considered significant at *P* < 0.05.

## Results

### Comparison between porcine ASCs isolated from two different body sites

**Figure 2:**
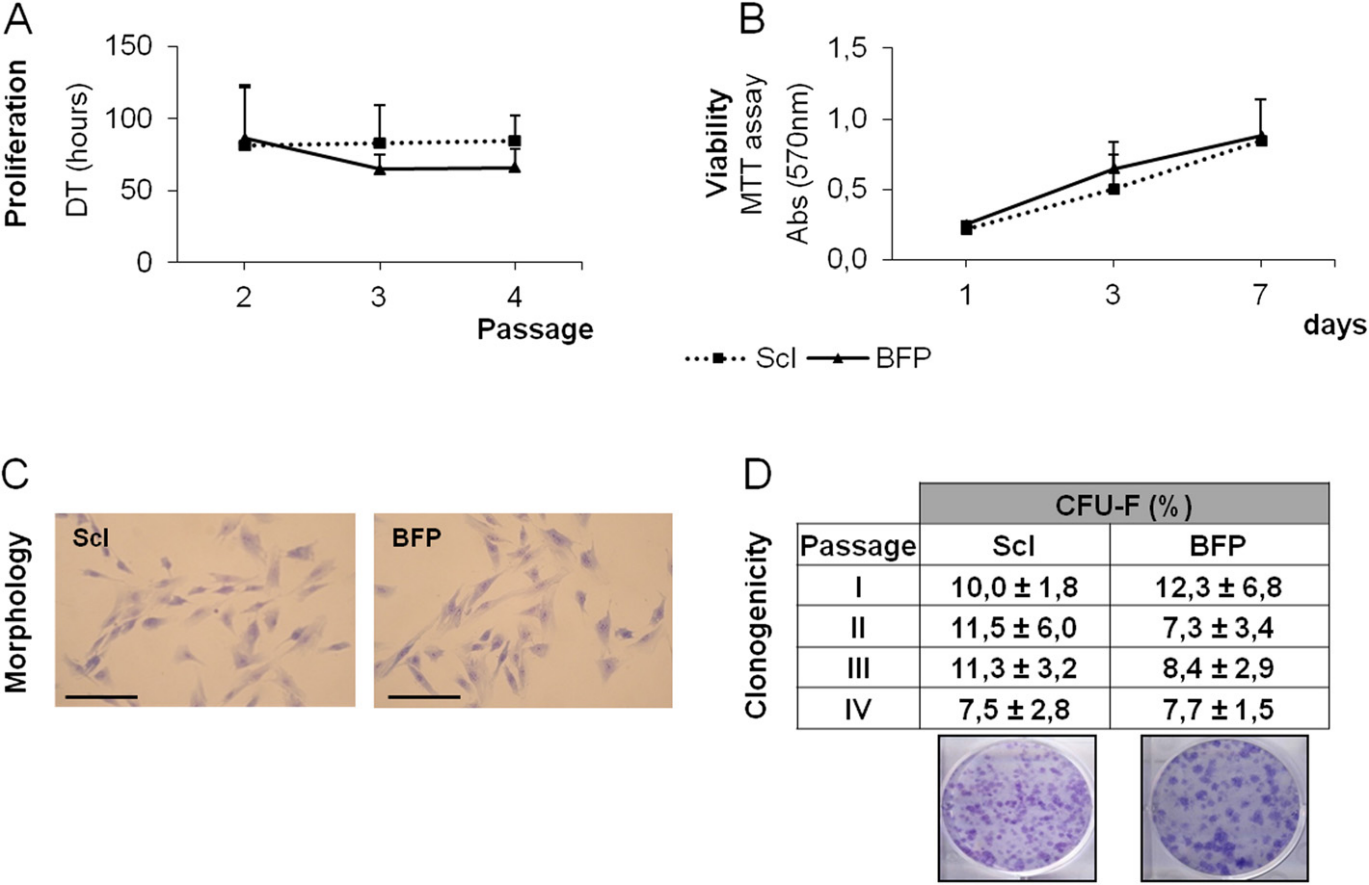
**Stemness features of ScI- and BFP-pASCs.** Cell proliferation expressed as doubling time (DT, hours) of ASCs from II to IV passage **(A)**. Viability assessed by MTT assay at days 1, 3, and 7 **(B)**. Morphology of ScI-pASCs and BFP-pASCs (optical microscopy, 200× magnification; scale bar, 100 μm) **(C)**. Clonogenicity from passage I to IV expressed as colony-forming units (CFU-F) percentage (ratio of number of colonies/number of plated cells × 100) (**D**, upper panel). Data are expressed as mean ± SEM (*n* = 6). Representative ScI-pASCs and BFP-pASCs plates stained with crystal violet (**D**, lower panel).

We collected different amounts of subcutaneous interscapular adipose tissue (ScI-pASCs) and buccal fat pad (BFP-pASCs) from six swine, as indicated in Table 1. We isolated 5.5 × 10^4^ ± 3.3 × 10^4^ ScI-pASCs/ml and 3.0 × 10^4^ ± 9.3 × 10^3^ BFP-pASCs/ml of raw tissue. pASCs adhered nicely to tissue-culture plates, and in a week, they began to proliferate, showing an MSC-typical fibroblast-like morphology (Figure 2C). In details, the doubling times (DT) of the two cell populations were constant, and no significant differences were observed between ScI-pASCs and BFP-pASCs. Indeed, the mean DT was of about 82.9 ± 11.5 hours for ScI-pASCs and 72.5 ± 8.2 hours for BFP-pASCs (Figure 2A). Furthermore, cell viability was maintained for all the pASC populations analyzed (Figure 2B), and their proliferation trend was quite stable.

**Table 1:** pASC source

		**Gender**	**Age**	***n***	**Raw adipose tissue (ml)**
**pASCs**	**ScI**	3 ♂, 3 ♀	≥4 months	6	12.3 ± 3.6
	**BFP**	3 ♂, 3 ♀	≥5 months	6	5.7 ± 1.5

Gender, age of the animals, amount of harvested fat from subcutaneous interscapular adipose tissue (ScI), and buccal fat pad (BFP). Data are expressed as mean ± SEM.

**Figure 3:**
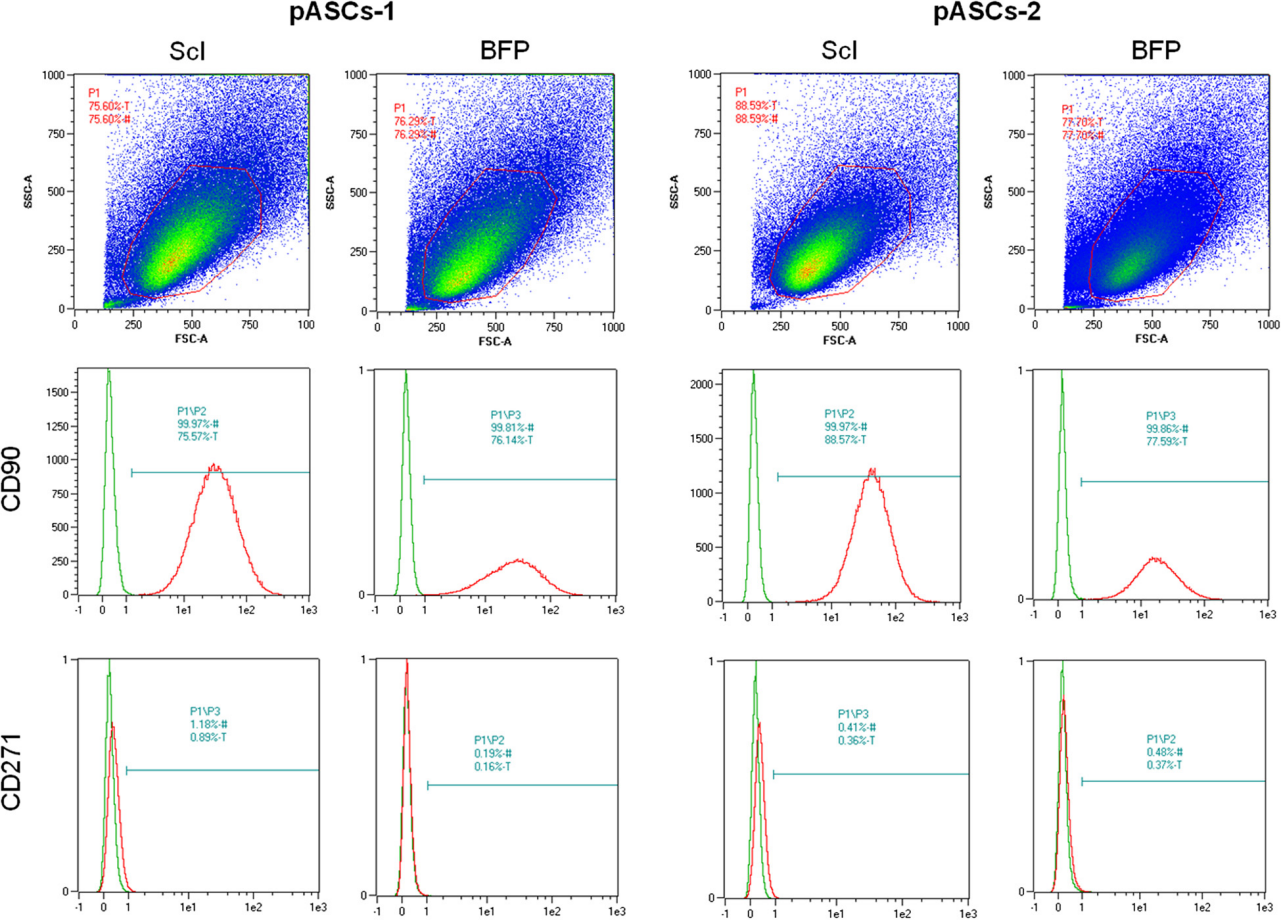
**FACS analysis of ScI- and BFP-pASCs.** Expression of specific mesenchymal stem cell markers in ScI- and BFP-pASC populations (*n* = 2). Size and granularity are shown (upper panels). pASCs stained for CD90 and CD271 are reported (lower panels).

Porcine ASCs held a strong clonogenic ability that was maintained along passage I to IV (Figure 2D): about 10.1% ± 1.4% of ScI-pASCs and 8.9% ± 1.5% of BFP-pASCs produced CFU-F. Moreover, both pASC populations were immunophenotyped, and a FACS analysis of both cells derived from two animals is shown in Figure 3. Both ScI- and BFP-pASCs appeared similar in size and granularity (upper panels), and both cell types expressed CD90 (middle panels), whereas the CD271 was not detectable (lower panels), as CD14 and CD45 (data not shown). Unfortunately, no cross-reactivity was found on both ScI- and BFP-pASCs for CD73 and CD105 (data not shown).

### Osteogenic, adipogenic, and chondrogenic differentiation of ScI-pASCs and BFP-pASCs

**Figure 4:**
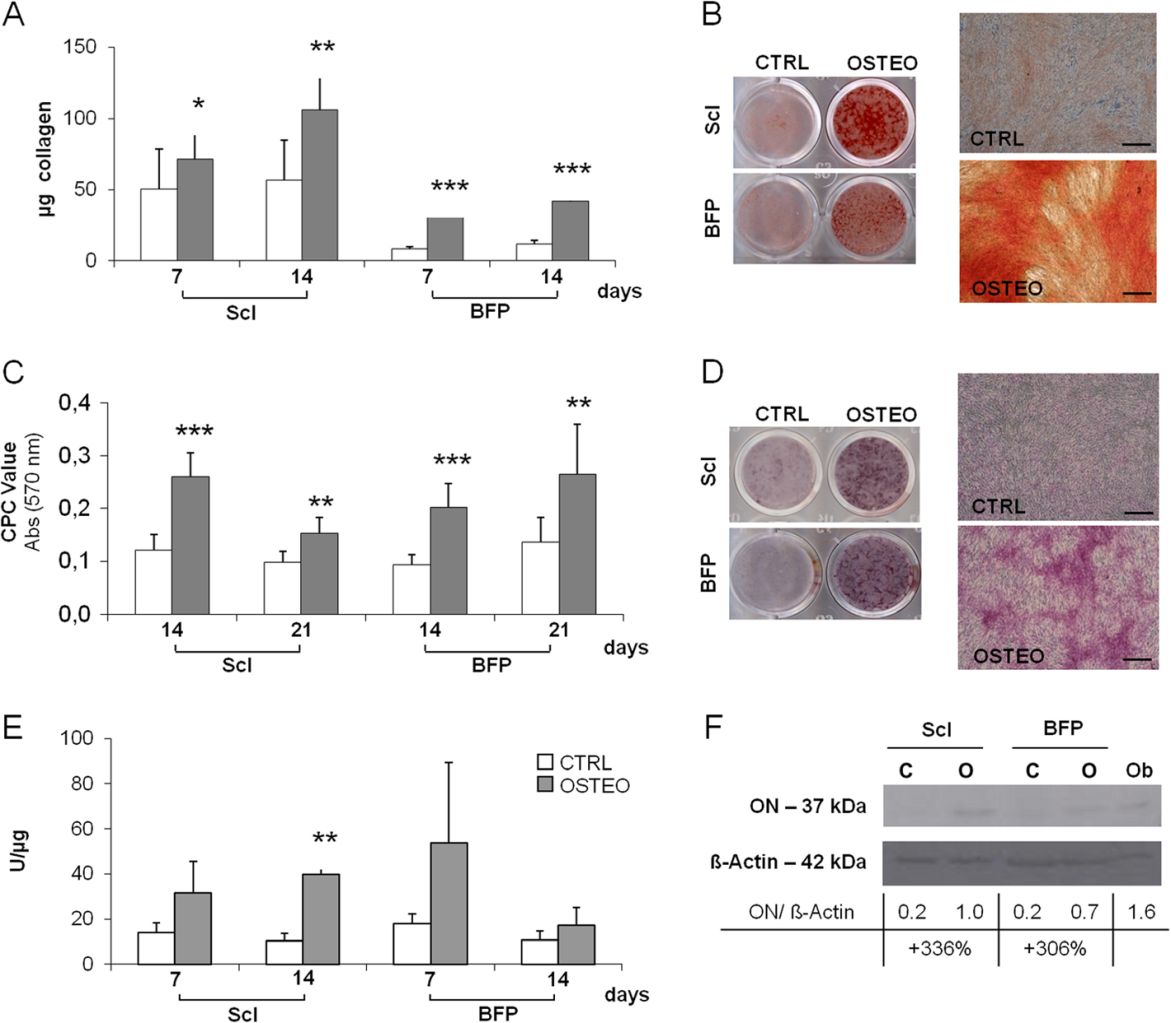
**Osteogenic potential of pASCs.** Quantification of collagen **(A)**, calcified extracellular matrix (ECM) deposition **(C)**, alkaline phosphatase (ALP) activity **(E)** in undifferentiated (CTRL, white bars), and osteo-differentiated (OSTEO, dark bars) ScI- and BFP-pASCs; data are expressed as mean ± SEM (*n* = 12). OSTEO versus CTRL *P < 0.05; **P < 0.01; ***P < 0.001. Images of ScI- and BFP-pASC wells stained with Sirius Red (**B**, left panel) and Alizarin Red-S (**D**, left panel) and representative microphotographs of ScI-pASCs (**B**, **D**, right panel, optical microscopy, 40× magnification; scale bar, 200 μm). Osteonectin expression of ScI-pASCs and BFP-pASCs analyzed with Western blot; its quantification, normalized to β-actin, is also indicated **(F)**.

Osteogenic differentiated ScI- and BFP-pASCs significantly increased the production of bone-specific markers, such as collagen (Figure 4A,B), calcified ECM (Figure 4C,D), alkaline phosphatase (ALP) activity (Figure 4E), and osteonectin (ON, Figure 4F), compared with undifferentiated cells.

Although BFP-pASCs showed a slightly lower basal level of collagen deposition with respect to ScI-pASCs, these differences at 7 and 14 days were not significant. Collagen level significantly increased by about 42% and 310% in 7 days osteoinduced ScI- and BFP-pASCs, respectively, compared with undifferentiated cells (CTRL); this upregulation further increased for ScI-pASCs (+87%) and was maintained (+254%) for BFP-pASCs (Figure 4A,B). In addition, osteodifferentiated pASCs produced abundant amounts of calcified ECM, and in 2 weeks, ECM calcification increased by about 118% and 116% for ScI- and for BFP-pASCs, respectively (Figure 4C,D). Meanwhile, ALP activity was also determined: after 1 week of culture, we observed an upregulated ALP activity in both osteo-differentiated ScI and BFP cells, compared with undifferentiated ones, with increases of 126% and 201%, respectively (Figure 4E). This trend was also maintained after 14 days (Figure 4E). In Figure 4F, we show that osteonectin (ON) expression is induced of about 336% and 306% in osteodifferentiated ScI- and BFP-pASCs, respectively.

**Figure 5:**
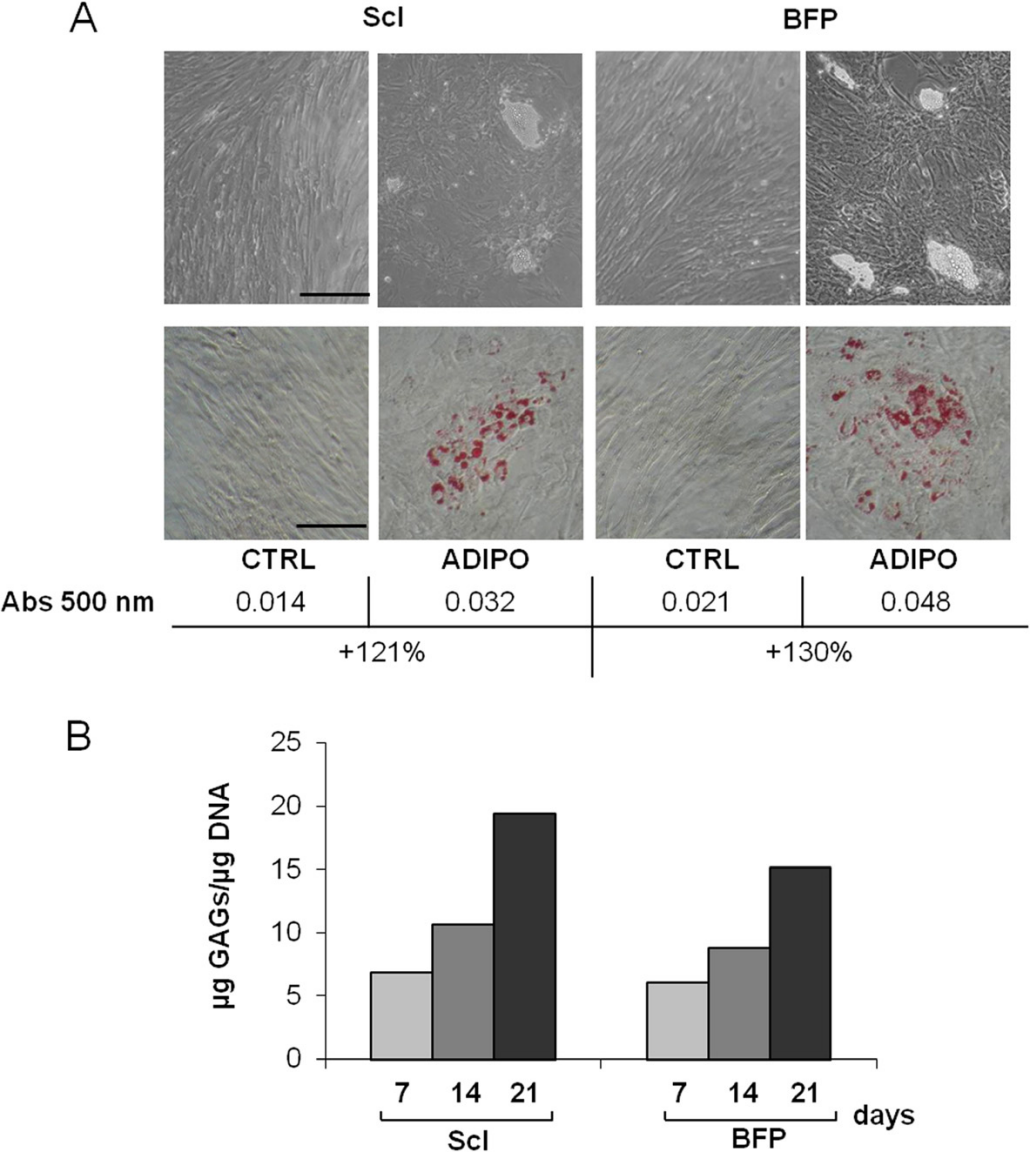
**Adipogenic and chondrogenic potential of pASCs.** Microphotographs of BFP-pASCs and ScI-pASCs maintained for 14 days in control (CTRL) and adipogenic medium (ADIPO; 200× magnification; scale bar, 50 μm), both during culture (**A**, upper panels) and after lipid vacuoles staining by Oil Red O (**B**, middle panels). Quantification of lipid vacuoles formation by Oil Red O extraction is shown in **A**, lower panel. Quantification of glycosaminoglycans (GAGs) production normalized on DNA content in CHONDRO-pASCs after 7, 14, and 21 days of differentiation in pellet culture **(B)**.

The multidifferentiative ability of BFP-pASCs was further tested and compared with ScI-pASCs. At first, as shown in Figure 5A, adipogenic differentiation is observed after 14 days: the morphology of both cell types is remarkably modified, from the usual fibroblast-like shape to a round one, with a cytoplasmic accumulation of lipid vacuoles (upper panel). Oil Red O staining (Figure 5A, middle panel), and its quantification proved that both cell types similarly differentiate (+121% for ScI-pASCs and +130% for BFP-pASCs, with respect to control cells) (Figure 5A, lower panel).

Then we also determined GAGs content in both chondrogenic differentiated pASCs for 1, 2, and 3 weeks. We observed an increase of GAGs deposition during that time. Indeed, after 14 days, the GAG content, with respect to 7 days, was more abundant at 56% and 45% in ScI- and BFP-pASCs, respectively, and it was further upregulated after 21 days (+184% and +149% for ScI- and BFP-pASCs, respectively) (Figure 5B). We conclude that both pASCs display *in vitro* the multipotent feature of mesenchymal stem cells.

### pASCs on biomaterials

**Figure 6:**
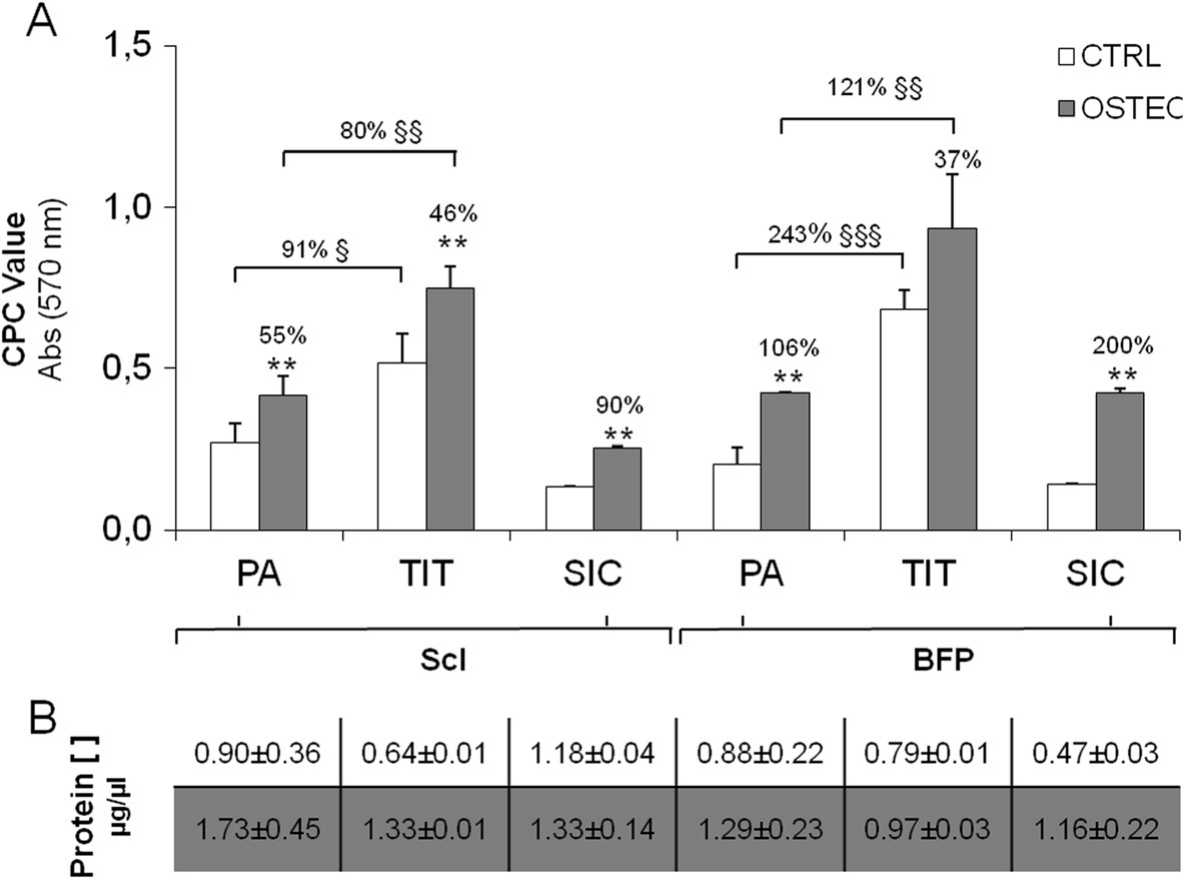
**Influence of biomaterials on pASC osteogenic differentiation.** Calcified ECM deposition in undifferentiated (CTRL, white bars) and osteogenic-differentiated (OSTEO, dark bars) ScI- and BFP-pASCs, cultured for 21 days on monolayer (plastic adherent, PA), or seeded on titanium disks (TIT) or on silicon carbide–plasma-enhanced chemical vapor deposition (SIC) fragments **(A)**. Protein concentration of pASCs cultured either in monolayer or adhering to biomaterial both in CTRL (white row) and OSTEO (dark row) is reported in panel **(B)**. Data are expressed as mean ± SEM (*n* = 3). OSTEO versus CTRL: **P < 0.01; TIT versus PA; ^§^*P* < 0.05; ^§§^*P* < 0.01; ^§§§^*P* < 0.001.

pASCs cultured for 21 days on biomaterials, both in the presence and in the absence of osteogenic stimuli, efficiently adhered to them; indeed, no significant differences were observed between the protein concentrations of either plastic adherent cells or scaffold-associated ones. This indirect evidence is shown in Figure 6B. ScI- and BFP-pASCs cultured for 7 days on the supports are alive and tightly laid on them when observed by confocal microscopy (data not shown).

Both pASCs, cultured on biomaterials, differentiated toward cells of the osteogenic lineage. Indeed, pASCs seeded on TIT, and osteodifferentiated, deposited an increased amount of calcified ECM of about 46% and 37% for ScI- and BFP-pASCs, respectively, compared with CTRL cells; similarly, ScI- and BFP-pASCs on SIC, increased ECM deposition of 90% and 200%, respectively, compared with CTRL cells.

Moreover, TIT is osteoinductive for pASCs; we quantified an increase of calcified ECM of about 91% in CTRL ScI-pASCs, and of about 234% in CTRL BFP-pASCs, compared with plastic-adherent cells (Figure 6A).

### Culture of pASCs in porcine serum

**Figure 7:**
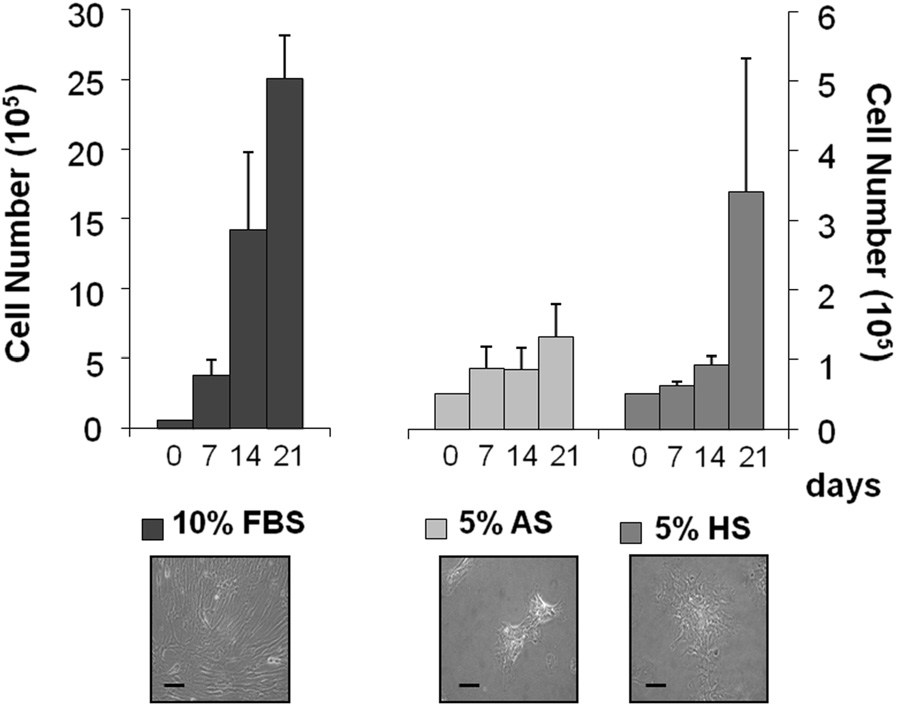
**pASCs cultured in media supplemented with porcine sera.** pASCs were grown for 7, 14, and 21 days, in DMEM supplemented with 10% FBS or 5% autologous or heterologous serum. Data are expressed as mean ± SEM (*n* = 4). Microphotographs of ScI-pASCs in culture for 21 days (lower panel, optical microscopy, 100× magnification, scale bar 50 μm). AS, autologous serum; FBS, fetal bovine serum; HS, heterologous serum.

Considering porcine ASCs useful in preclinical models, we compared their behavior when they were cultured in medium supplemented with porcine serum, 5% autologous (AS), and 5% heterologous sera (HS), and with 10% FBS (standard condition). Cells did not proliferate as fast as when maintained in standard condition. In more detail, the number of pASCs collected after 3 weeks was about 4.4% ± 2.4% compared with cells grown in standard condition and set as 100% (data not shown). After 7 days, the presence of either autologous or heterologous sera did not allow a rapid cell growth. However, after 21 days, pASCs grown in the presence of HS increased their number with respect to pASCs cultured in AS (Figure 7). As depicted also in Figure 7, all ASCs, grown in the presence of autologous or heterologous serum, aggregated in small clusters and changed morphology, becoming smaller and rounder compared with cells cultured in 10% FBS.

## Discussion

We investigated the possibility of isolating porcine ASCs from Buccal Fat Pads (BFP-pASCs), which have similar stemness features to the ones isolated from subcutaneous tissue (ScI-pASCs), previously characterized [34]. Human BFP-ASCs might be quite easily applied in oral tissue engineering, because this tissue is rapidly accessible by dentists and maxillofacial surgeons [14]. However, before moving to the clinic, it is mandatory to perform approved preclinical studies to validate the safety and efficacy of cellular therapies. The most used large-animal model of human oral bone defects is swine [31,42], because these animals present a healing potential comparable to that of the human. Several studies have been conducted by using stem cells in oral diseases and orofacial research: Wilson *et al*. [31] investigated bone regeneration in the pig mandible ramus by either local or systemic ASCs injection, concluding that both treatments accelerate the healing process, without any significant difference between the two routes of administration. In another study, similar results were obtained combining decidua stem cells with a β-TCP scaffold in a minipig model [43].

Here we compared pASCs derived from two different body areas and evaluated their behavior *in vitro* to identify a convenient source for future preclinical studies. BFP-pASCs were very similar to ScI-pASCs. Although the cellular yield of the porcine ASCs was lower than the human one [44], after 30 days in culture, we could have been able to obtain a homogeneous populations of about 10^8^ to 10^9^ cells, with still a pronounced clonogenic ability.

Both cell populations, analyzed at passage 4, were CD90^+^, CD271^-^, CD45^-^, and CD14^-^. These results are similar to the ones on porcine MSCs from different tissues [45], and to our results on human mesenchymal stem cells from the Bichat fat pad that express CD90, CD73, and CD105 [21], as defined for human mesenchymal stromal cells [35,36].

In conclusion, both cell populations were highly positive for CD90, one of the main MSC surface antigens, whereas no cross-reactivity has been observed for CD73 and CD105. Although limited, these results are consistent with the ones obtained with porcine MSCs from bone marrow [46].

Furthermore, by a molecular approach of RT-PCR, we have preliminary data on the expression of Kruppel-like factor 4 (Klf-4), a marker of immature stem cells involved in the control of cell multipotency in many development-related processes and in the maintenance of stem cell-associated properties [47]. The mRNA expression levels in BFP-pASCs are comparable to the ones in human-ASCs. We consider this result interesting, because we recently showed that Klf-4 expression in hASCs seems to be related to the cell proliferation, clonogenic ability, and differentiative potential, and to be downregulated by the pathologic condition (obesity) of patients from which cells were isolated [48].

Besides, all the porcine BFP-ASCs, grown in the presence of inductive stimuli, nicely increased both osteogenic and adipogenic features, as already described for subcutaneous porcine ASCs [34,49,50]. At last, both populations are able to progressively depose GAGs during 3D culture when induced to chondro-differentiate. Altogether, these results suggest our claim that swine buccal fat pad contains progenitor cells of the mesenchymal stromal cell family, similar to the human ones.

Because these cells could be used in preclinical studies of tissue engineering, and their interaction with appropriate supports is essential, we evaluated the ability of both pASCs to grow and differentiate onto two synthetic scaffolds: the former, a widely used biomaterial in dental surgeries (titanium), and the latter, a promising candidate for the coating of some portions of implant (SiC-PECVD). Like human ASCs [32], pASCs adhere and differentiate on both scaffolds. Moreover, the osteoinductive properties of titanium on hASCs [32], were also observed on both porcine progenitor cells, whereas SIC-PECVD did not modulate their osteogenic differentiation.

Next, testing porcine autologous or heterologous sera, we detected that pASCs proliferated slower than cells cultured in the presence of FBS, and they dramatically stopped growing, changed morphology, and aggregated in clusters. These data are consistent with previous data by Schwarz *et al*. [41], in which equine ASCs cultured with autologous serum proliferate less than with FBS. Differently, our results are in contrast with data obtained with human ASCs, in which it has been shown that the use of autologous serum favors or does not influence ASCs proliferation [21,51,52]. Nevertheless, Kurita *et al.*[53] showed that among four human ASC populations, only one proliferates faster when cultured with autologous serum. This discrepancy has also been observed for human bone marrow stem cells [54-57], suggesting that other factors may influence cell growth. This issue requires further investigation to be clarified, although we have shown that both pASCs behaved similarly.

## Conclusions

Our data suggest that the buccal fat pad might be a novel source of MSCs. This region contains a population of progenitor cells with stemness features that are able to differentiate *in vitro* and also are associated with synthetic supports. This is quite relevant for maxillofacial and dental surgeons, because for them, human BFP is an easily reachable and convenient area. Human ASCs have been isolated from small specimens of BFP (1 ml of tissue), and they are similar to the most known ScI-ASCs [21,58]. Data about human cells and previous data on pASCs [2,8,34] are consistent with our work, indicating that BFP-ASCs are comparable with ASCs isolated from human and porcine subcutaneous tissue. Although it could be debatable to isolate cells from a very small fat pad, we think that progenitor cells derived from a nearby area of the defect could push toward a proper use of BFP-ASCs in oral clinical studies. The natural localization of BFP-ASCs could make them more prone to respond to stimuli naturally secreted in the mouth, as we previously observed regarding the osteoinductive properties on human BFP-ASCs of amelogenin, the most abundant enamel matrix protein [21]. For preclinical test, the low immunogenicity of mesenchymal stem cells might be exploited by using heterologous porcine BFP-ASCs, because in swine, the buccal fat pad is not so easily accessible as in the humans.

Our data support future clinical applications of human BFP-ASCs in a tissue-engineering approach for oral and maxillofacial diseases, and we suggest swine as a convenient preclinical model to test new bioconstructs.

## Abbreviations

AR-S: Alizarin red-s; AS: Autologous serum; ASCs: Adipose-derived stem cells; BFP: Buccal fat pad; CFU-F: Fibroblast-colony-forming unit; CPC: Cetylpyridinium chloride; CTRL: Control medium; DMEM: Dulbecco modified Eagle medium; DMSO: Dimethyl sulfoxide; DT: Doubling time; ECM: Extracellular matrix; EDTA: Ethylenediaminetetraacetic acid; FBS: Fetal bovine serum; HS: Heterologous serum; MSCs: Mesenchymal stem cells; MTT: 3-[4,5 dimethylthiazol-2-yl]-2,5-diphenyltetrazolium bromide; OSTEO: Osteogenic medium; ScI: Subcutaneous interscapular; SEM: Standard error of the mean; SIC: Silicon carbide; SIC-PECVD: Silicon carbide–plasma-enhanced chemical vapor deposition; SVF: Stromal vascular fraction; TIT: Titanium.

## Competing interests

The authors declare that they have no competing interests.

## Authors’ contributions

SN and LMF participated in the conception and design of the study, collecting samples, and their analysis and interpretation. They carried out all the cell isolation, expansion, and characterization. They carried out FACS and biochemical analyses, and gene and protein expression tests. Both authors have been involved in the writing process, and their critical intellectual role has been determinant. EA made substantial contributions to acquisition, analysis, and interpretation of data, and she carried out the statistical analysis. She has also been involved in manuscript writing. AA made substantial contributions to design, analysis, and interpretation of data, and she was responsible of animal handling and surgery. MC has made substantial contributions to the *in vivo* part of the study and in the *in vivo* study design, collecting biologic specimens, acquiring data, and their interpretation. EB contributed to the conception and design of the study, and interpretation of the data and he was involved in drafting the manuscript and revising it critically for important intellectual content. ATB contributed to conception and design of the study, analysis and interpretation of the data, and she was involved in drafting the manuscript and in the study’s funding. She also gave final approval of the version to be published, and she agreed to be accountable for all aspects of the work in ensuring that questions related to the accuracy or integrity of any part of the work are appropriately investigated and resolved. All authors read and approved the final manuscript.
